# Interprofessional education in cardiothoracic surgery: a narrative review

**DOI:** 10.3389/fsurg.2024.1467940

**Published:** 2024-09-04

**Authors:** Savvas Lampridis, Marco Scarci, Robert J. Cerfolio

**Affiliations:** ^1^ National Heart and Lung Institute, Faculty of Medicine, Imperial College London, London, United Kingdom; ^2^ Department of Thoracic Surgery, 424 General Military Hospital, Thessaloniki, Greece; ^3^ Department of Cardiothoracic Surgery, Hammersmith Hospital, London, United Kingdom; ^4^ Department of Cardiothoracic Surgery, New York University Langone Health, New York, NY, United States

**Keywords:** cardiothoracic surgery, curriculum, interprofessional collaboration, interprofessional education, multidisciplinary team, simulation, surgical education

## Abstract

Interprofessional education, an approach where healthcare professionals from various disciplines learn with, from, and about each other, is widely recognized as an important strategy for improving collaborative practice and patient outcomes. This narrative review explores the current state and future directions of interprofessional education in cardiothoracic surgery. We conducted a literature search using the PubMed, Scopus, and Web of Science databases, focusing on English-language articles published after 2000. Our qualitative synthesis identified key themes related to interprofessional education interventions, outcomes, and challenges. The integration of interprofessional education in cardiothoracic surgery training programs varies across regions, with a common focus on teamwork and interpersonal communication. Simulation-based training has emerged as a leading modality for cultivating these skills in multidisciplinary settings, with studies showing improvements in team performance, crisis management, and patient safety. However, significant hurdles remain, including professional socialization, hierarchies, stereotypes, resistance to role expansion, and logistical constraints. Future efforts in this field should prioritize deeper curricular integration, continuous faculty development, strong leadership support, robust outcome evaluation, and sustained political and financial commitment. The integration of interprofessional education in cardiothoracic surgery offers considerable potential for enhancing patient care quality, but realizing this vision requires a multifaceted approach. This approach must address individual, organizational, and systemic factors to build an evidence-based framework for implementation.

## 1 Introduction

Interprofessional education is widely acknowledged as a pivotal strategy for developing collaborative practice and improving health outcomes across several medical specialties (1–3). This educational approach, where healthcare professionals from diverse disciplines learn about, from, and with each other, prepares medical practitioners to deliver safe and patient-centered care (4, 5). At its core, interprofessional education aims to dissolve professional barriers, nurture mutual respect, and build cohesive healthcare teams through shared learning experiences (6, 7). Such a model is necessary in today's healthcare landscape, where complex patient needs demand a comprehensive and coordinated approach spanning various health disciplines (8, 9).

Interprofessional education gained momentum after the publication of multiple patient safety studies that highlighted the significance of collaborative practice (10–12). In recent years, policymakers and educational leaders have increasingly advocated for the widespread integration of interprofessional education into medical curricula, emphasizing its potential to improve patient outcomes and satisfaction (13). Prominent international organizations, including the World Health Organization, the World Federation of Medical Education, and the Organization for Economic Co-operation and Development, have proposed strategies to promote interprofessional education in healthcare (5, 14).

In cardiothoracic surgery, the need for interprofessional education is amplified due to the complicated nature of procedures and the multidisciplinary approach required for patient management. Over the past two decades, this specialty has witnessed significant technological advancements, such as robotic surgery and transcatheter techniques. These innovations require an educational framework that not only teaches technical expertise but also cultivates competencies in communication and teamwork (15–19). Furthermore, the rapid evolution of thoracic oncology, with its expanding array of anticancer treatments, necessitates close cooperation between thoracic surgeons and specialists from various disciplines (20–22). Moreover, collaborative competencies are needed to address challenges arising from perioperative care pathways for cardiothoracic surgery patients (23). Interprofessional education can improve professional interactions in these and many other settings involving cardiothoracic surgeons.

While the effectiveness of interprofessional education is well-established in general healthcare settings, its specific outcomes, benefits, and challenges in cardiothoracic surgery remain inadequately explored. This review aims to synthesize available evidence and outline strategies for integrating interprofessional collaboration in cardiothoracic surgery education.

## 2 Methods

We performed a literature search using the PubMed, Scopus, and Web of Science databases on April 1, 2024. To capture recently published material, we enabled auto-alerts through May 12, 2024. The search strategy included the following keywords: interprofessional education, cardiothoracic surgery, collaborative practice, multidisciplinary team, and surgical education. We limited the results to English-language articles published after 2000, without restrictions on study design. The initial screening process involved reviewing titles and abstracts to exclude articles clearly unrelated to our topic, such as those focusing solely on technical skills. We then obtained and evaluated the full-text versions of the remaining articles. Data from the selected studies were synthesized narratively, with a focus on interprofessional education interventions, outcomes, barriers, and facilitators. As this review did not involve the collection of primary patient data, ethical approval was not necessary. In conducting this literature review, we adhered to the guidelines outlined in the Scale for the Assessment of Narrative Review Articles (24).

## 3 Status of interprofessional education

Historically, cardiothoracic surgery training has primarily focused on developing the technical skills essential for performing a predefined scope of procedures (25–27). Mastering these skills is demanding given the high precision required for surgical interventions on vital intrathoracic organs. In recent years, the educational landscape of cardiothoracic surgery has undergone a substantial transformation, evolving in response to advances in medical knowledge, technological innovations, and changing societal needs (28, 29). This shift marks the beginning of an endeavor to define an educational framework that addresses not only the requisite technical proficiencies but also the behavioral skills necessary for team-based care.

The curriculum and structure of cardiothoracic surgery training vary widely across different regions and healthcare systems, each adapting to the unique needs of their medical and regulatory environments. As a common denominator, however, there has been a growing interest in non-technical skills. In the United States, the American Board of Medical Specialties and the Accreditation Council for Graduate Medical Education have jointly approved core competencies that provide a framework for important developmental areas. Among these, “interpersonal and communication skills” stands out as a core competency that contains the subcomponent of interprofessional and team communication (30, 31).

The United Kingdom has taken a similar approach to integrating interprofessional education in cardiothoracic surgery training. The country's training framework emphasizes capabilities in practice that are shared across all surgical specialties, with multidisciplinary team management as a key component (32). Moreover, the professional behaviors and competencies assessed during training are aligned with the General Medical Council's “Generic Professional Capabilities Framework” (33). This framework includes interpersonal and communication skills, ensuring that non-technical skills receive appropriate attention throughout the training program.

The European Board of Cardiothoracic Surgery is working towards standardizing training and promoting mutual recognition of qualifications across European Union member states. During assessment, candidates must demonstrate specific professional behaviors, including application of the principles of multidisciplinary and team-based care and selection of effective communication strategies to maintain patient safety and reduce the risk of medical errors (34). The thoracic oncology HERMES (Harmonizing Education in Respiratory Medicine for European Specialists) curriculum exemplifies the integration of interprofessional education in thoracic oncology training (35). This program advocates for the indispensable role of multidisciplinary teams in treating thoracic malignancies by promoting collaborative decision-making processes throughout all stages of cancer care. Various specialists, including thoracic surgeons, medical oncologists, radiation oncologists, and palliative care experts, are expected to seamlessly work together in order to achieve optimal patient outcomes. Interprofessional collaboration is promoted through structured educational activities and is a mandatory requirement for certification.

Numerous institutions worldwide have recognized the importance of early interprofessional education and have implemented programs at the undergraduate level. For instance, the University of British Columbia in Canada has been a pioneer in integrating interprofessional and interdisciplinary education into its health and human service programs over the past few decades (36). In the United Kingdom, interprofessional education is now a standard component in pre-qualifying health and social care courses in at least two-thirds of relevant universities (37). An increasing interest in the integration of interprofessional education in undergraduate medical curricula has also been documented in other countries within the European Higher Education Area, with Germany and Sweden as notable examples (38). These widespread initiatives demonstrate a growing global recognition of the value of early exposure to interprofessional collaboration in medical education. However, it is important to note that despite this progress, significant challenges and limitations continue to exist in the implementation and standardization of these programs across different educational systems and cultural contexts (38, 39).

## 4 Impact of interprofessional education

Interprofessional education has the potential to improve various aspects of cardiothoracic surgery, from patient care and safety to medical education and research (Table 1). Non-technical skills, such as communication, teamwork and collaboration, are important for the smooth functioning of multidisciplinary teams in complex surgical environments. Surgical teams that exhibit strong social skills have been associated with improved patient outcomes and lower mortality rates (47–49). Conversely, breakdowns in communication and teamwork have been linked to adverse events, preventable errors, and increased mortality rates (50, 51).

**Table 1:** Summary of studies on interprofessional education outcomes in cardiothoracic surgery.

Authors, year	Country	Participants	Design	Intervention	Findings
Bierer et al., 2018 (40)	Canada	Thoracic surgery fellows, thoracic surgeons, nurses, anesthesia staff	Operating room simulation	Airway obstruction by residual tumor after pneumonectomy	• Demonstrated construct validity in differentiating non-technical skill levels • Identified potential latent safety threats • Showed high fidelity and educational value as per participant feedback
Merritt-Genore et al., 2022 (41)	USA	Cardiothoracic surgery fellows, cardiac anesthesia fellows, perfusionists, general surgery residents, anesthesiology residents, operating room staff	High-fidelity lab simulation	Critical steps and potential crises during cardiopulmonary bypass	• Improved participant self-confidence, especially in managing iatrogenic dissection and emergent return to cardiopulmonary bypass • Improved multidisciplinary perspective and team performance • Participants requested further similar events
Baste et al., 2021 (42)	France	Surgeons, surgery fellows and residents, anesthesiologists, anesthesiology fellows and residents, nurses, operating room technicians, medical students	Hybrid, medium-to-high fidelity simulation	Life-threatening events (e.g., bleeding, contralateral tension pneumothorax) in robotic and video-assisted thoracic surgery	• Improved team performance and patient safety • Received positive feedback from team members
Burkhart et al., 2013 (43)	USA	Cardiothoracic surgery residents, cardiothoracic surgeons, anesthesiologists, perfusionists	Didactic lectures and lab simulation	Identification of components post-cardiotomy extracorporeal membrane oxygenation circuit and management of crisis scenarios	• Improved residents’ knowledge, confidence, and crisis management skills • Most residents strongly recommended the course to their peers
Kemper et al., 2023 (44)	Netherlands	Cardiac anesthesiologists, cardiac surgeons, perfusionists, operating room nurses	Delphi method	National survey to identify crisis scenarios for simulation-based team training in cardiac surgery	• Identified 13 scenarios with an expert consensus >67% • Highlighted the value of interprofessional consensus in tailoring simulation curricula
Meyenfeldt et al., 2022 (45)	Netherlands	General thoracic and cardiothoracic surgeons, anesthesiologists, pulmonary physicians, nurses, healthcare managers, patient representatives, electronic medical record specialist, healthcare insurance company representative	Semi-structured interviews	National survey to identify determinants for enhanced recovery program after lung cancer surgery	• Facilitators included clear multidisciplinary protocol, leadership from a senior clinician, support from a program coordinator, and patient involvement in decision-making processes • Inconsistent communication between team members was a barrier
Allen et al., 2009 (46)	USA	First-year medical students, cardiothoracic surgeons, laboratory residents, faculty mentors	Cardiothoracic surgery clinic, operating room, and laboratory	8-week program with surgeon shadowing, large-animal operations, and research project	• Increased academic productivity • Guided 80% of eligible students toward surgical specialties, including cardiothoracic surgery

Simulation-based interprofessional education is an effective method for teaching these non-technical skills (52, 53). This approach provides a safe and controlled environment where healthcare professionals from different disciplines can collaboratively practice teamwork, communication, and crisis management, without putting patients at risk (54). Importantly, concomitant technical skills training, such as simulating operative procedures, has been shown to significantly improve the acquisition of non-technical competencies (55, 56). This finding supports the notion that technical and non-technical skills are intrinsically linked and should be learned together, reflecting the challenges of real-world clinical settings (57).

The feasibility and effectiveness of interprofessional simulation-based training in the cardiothoracic operating room have been demonstrated by multiple studies. Bierer et al. (40) described the development and implementation of an inexpensive, *in situ* simulation model for training non-technical skills in managing an intraoperative airway crisis following pneumonectomy. This simulation scenario brought together participants from surgery, anesthesia, and nursing, creating an environment that closely resembled real-world operating room dynamics. Evaluations using validated instruments, such as Non-Technical Skills for Surgeons (58) and TeamSTEPPS2 (59), confirmed the model's construct validity by distinguishing non-technical skill levels between consultant surgeons and surgical trainees. Furthermore, the simulation revealed potential latent safety threats, including missing equipment and knowledge gaps regarding team members’ roles during a crisis. Participant feedback via the Method Material Member Overall questionnaire corroborated the high fidelity and educational value of the simulation experience. The success of this immersive, low-cost model shows its potential as a readily adoptable approach for institutions with limited simulation resources. It can empower thoracic surgery trainees to refine their non-technical skills while proactively identifying and addressing system vulnerabilities to improve patient safety practices.

In a similar study from the field of cardiac surgery, Merritt-Genore et al. (41) developed high-fidelity simulation events that replicated critical steps and potential crises during cardiopulmonary bypass. The study involved participants from various disciplines, including cardiothoracic surgeons, anesthesiologists, and perfusionists. These scenarios were designed to promote team communication, familiarize participants with uncommon but high-stakes events, and increase confidence in managing such situations. The results showed a significant improvement in participants’ self-confidence when handling scenarios of iatrogenic dissection and emergent return to cardiopulmonary bypass. Additionally, subjective observations and participant feedback indicated an overall positive impact on team dynamics, understanding of multidisciplinary perspectives, and performance during simulated crises. By exposing participants to realistic, high-risk scenarios and rotating them through unfamiliar roles, the simulation facilitated the development of non-technical skills necessary in the cardiac operating room. This approach addresses the need for effective teamwork in settings where lapses can rapidly lead to severe adverse events.

Simulation-based training can also serve as a tool to equip operating room teams with the required skills for navigating the challenges posed by the adoption of novel surgical technologies. Minimally invasive thoracic surgery has been shown to improve patient outcomes compared with open surgical approaches (60). However, these advancements introduce challenges in coordinating personnel and technology, alter situational awareness, and create potential pitfalls (61). A retrospective analysis of patient safety incidents in the French national thoracic surgery database from 2008 to 2019 revealed that 65.6% of 407 events related to minimally invasive surgery were attributed to human factors (62). These incidents originated from the interplay between individuals, their tasks, and the workplace environment. This statistic illustrates the urgent need for continuous professional development and error management training within interprofessional frameworks that improve team dynamics and operational safety (63, 64).

Baste et al. (42) developed a simulation-based crisis training program utilizing realistic models of catastrophic events specific to minimally invasive thoracic surgery. Their hybrid model, featuring medium-to-high fidelity, immersed participants in stressful scenarios encountered during robotic and video-assisted thoracic surgery, such as uncontrolled bleeding and contralateral tension pneumothorax. The simulation underwent rigorous evaluation through debriefing sessions and an independent audit focusing on human factors. Positive feedback from team members and the program's demonstrable impact on patient safety and team performance suggest its significant value. Consequently, the authors advocate for the integration of team simulation training and crisis resource management into a continuous educational program for professionals in minimally invasive thoracic surgery.

Interprofessional crisis simulations have also shown value as educational tools in critical care settings. Burkhart et al. (43) developed and evaluated a clinical simulation program that trained cardiothoracic surgery residents in the principles and practical application of post-cardiotomy extracorporeal membrane oxygenation (ECMO). The two-day course, designed and facilitated by a multidisciplinary faculty including surgeons, anesthesiologists, and perfusionists, combined didactic lectures with hands-on simulation. The results indicated significant improvements in residents’ confidence, knowledge, and crisis management skills related to ECMO. Specifically, the residents’ ability to accurately identify ECMO components increased from 40% before training to 69% after the course. Additionally, their proficiency in managing simulated crisis scenarios, such as arterial desaturation and hypertension, showed substantial improvement. Overall, this multidisciplinary approach addressed key cognitive and behavioral competencies for ECMO management among cardiothoracic surgery residents.

Kemper et al. (44) employed a modified Delphi method to identify and reach consensus on crisis scenarios suitable for simulation-based, nontechnical skills training in cardiac surgery teams. The researchers surveyed 114 experts from various disciplines, including cardiac surgeons, anesthesiologists, perfusionists, and operating room nurses, identifying 13 highly relevant crisis scenarios for interprofessional simulation training. These scenarios included a range of critical events, such as cardiopulmonary bypass failure, emergency sternotomy, cardiac resuscitation, and massive bleeding. The study's strength lies in its inclusive approach, incorporating insights from all cardiac surgery team members. Each profession contributes unique perspectives on crisis situations based on their specific roles. Thus, interprofessional consensus is imperative for developing valid and clinically applicable scenarios for simulation-based curricula in cardiac surgery.

Interprofessional education can play a significant role in the successful implementation of enhanced recovery after thoracic surgery (ERATS) pathways. ERATS protocols have been shown to reduce postoperative complications, shorten hospital stays, decrease costs, and improve patient-reported outcomes (65). Meyenfeldt et al. (45) investigated the determinants for an ERATS pathway for Dutch lung cancer patients by conducting semi-structured interviews with a wide range of stakeholders. These included general thoracic and cardiothoracic surgeons, anesthesiologists, pulmonary physicians, nurses, healthcare managers, patient representatives, an electronic medical record specialist, and a healthcare insurance company representative. The findings indicate the importance of a clear and concise multidisciplinary protocol in ensuring consistent care delivery across the involved specialties. Other key facilitators included leadership from a senior clinician and support from an ERATS coordinator. In contrast, inconsistent communication was found to have a negative impact. Interestingly, providing patients with consistent information and involving them in decision-making processes were also identified as important factors for the successful application of the ERATS protocol. Interprofessional education, by fostering clear communication among diverse healthcare professionals, promoting leadership and coordination skills, and prioritizing patient-centered care, can significantly contribute to the effective implementation and success of ERATS pathways (66–68).

Integrating interprofessional education in cardiothoracic surgery has yielded benefits not only in residency programs but also in preparing medical students for collaborative healthcare environments. Allen et al. (46) implemented a structured program to introduce first-year medical students to cardiothoracic surgery and laboratory research. This three-part, eight-week program comprised shadowing a cardiothoracic surgeon in the outpatient clinic and operating room, participating in large-animal operations in the laboratory, and completing a clinical research project under the guidance of a laboratory resident and faculty mentor. The program resulted in significant academic output, with 18 participants producing 39 peer-reviewed manuscripts. Furthermore, it influenced 80% of the eligible students to pursue surgical specialties, including cardiothoracic surgery, for their residency. This program's outcomes suggest the potential benefits of early interprofessional experiences in cardiothoracic surgery education, aligning with current trends that advocate for early exposure to multidisciplinary roles in preparing students for the collaborative nature of modern healthcare (69, 70).

## 5 Implementing interprofessional education: challenges and strategies

Despite the well-documented benefits of interprofessional education in cardiothoracic surgery, its implementation remains challenging. Most obstacles arise from the complex nature of the specialty, deeply ingrained professional norms, and unique dynamics within surgical teams. Successfully integrating interprofessional education in training programs requires a systematic approach to identifying and addressing these challenges (Table 2).

**Table 2:** Challenges and strategies in implementing interprofessional education in cardiothoracic surgery.

Challenge	Description	Strategies to overcome challenge
Professional socialization	Deep sense of professional identity emphasizing individual expertise and autonomy in decision-making	• Socialization processes early in surgical training • Involvement of diverse specialists in joint simulations and case studies • Use of shared digital platforms for case discussions and procedural planning • Mentorship programs with senior professionals from different disciplines
Professional hierarchy	Hierarchical nature of medical professions leading to resistance in sharing critical decision-making roles	• Implementation of interprofessional communication frameworks early in surgical training • Encouragement of leadership roles among non-surgeon professionals • Mentorship programs to balance power dynamics • Creation of inclusive team identities to foster shared responsibility in patient outcomes
Professional stereotypes	Preconceived notions about roles, competencies, and educational levels of other professionals leading to tensions and inefficient teamwork	• Collaborative problem-solving tasks that provide opportunities for intergroup contact • Encouragement of leadership to model collaborative behaviors and actively promote interprofessional collaboration • Continuous professional development programs that reinforce interprofessional values and skills
Resistance to role expansion	Engaging in roles outside traditional scope can be met with fears of deskilling or overstepping professional boundaries	• Early exposure to roles and contributions of various healthcare professionals • Use of role theory to clarify expectations and boundaries • Structured programs with simulated scenarios to experience role expansion in a controlled environment • Leadership support and institutional policies to encourage role expansion
Logistical and administrative hurdles	Coordination challenges between multiple departments and specialties with different schedules, educational backgrounds, and priorities	• Establishment of a centralized faculty workgroup dedicated to coordination across departments • Alignment of academic calendars and creation of uniform scheduling blocks for interprofessional education activities • Investment in shared facilities, such as simulation labs and collaborative learning spaces • Financial and administrative support from organizations and institutions

### 5.1 Professional socialization

Professional socialization may present a significant barrier to implementing interprofessional collaboration, particularly in specialized fields like cardiothoracic surgery. This process involves the induction of new members into the norms, values, behaviors, and social knowledge of a profession (71, 72). For cardiothoracic surgeons, this often translates into a deeply ingrained sense of professional identity shaped through many years of highly specialized training (73). The intense focus on technical proficiency, individual expertise, and independent decision-making instilled during training reinforces a culture where collaborative practice may be perceived as a challenge to the cardiothoracic surgeon's authority or an encroachment on their autonomy. Modifying this entrenched professional identity is a critical first step in developing an environment where interprofessional collaboration is normalized and valued.

Socialization processes should begin early in medical education and surgical training. Many cardiothoracic surgery training programs are characterized by isolation, as evidenced by the physical separation of educational spaces and minimal cross-professional engagements. Altering this segregated educational model would encourage early exposure to and appreciation of the roles of various healthcare professionals (74). Involving physicians, nurses, and allied health professionals in joint simulations and case studies can help cardiothoracic surgeons learn to communicate effectively across disciplines, understand the scope of each team member's knowledge, and apply this collaborative approach to clinical settings. Additionally, shared digital platforms that facilitate case discussions and procedural planning between different specialties could support such interprofessional education efforts (75). Mentorship programs pairing cardiothoracic surgery residents with senior healthcare professionals from different disciplines can further promote interprofessional socialization. These mentors can model collaborative behaviors and demonstrate the value of interdisciplinary input in clinical decision-making (76, 77). Moreover, continuous professional development opportunities focusing on interprofessional collaboration should be offered throughout residency programs. Workshops, seminars, and interprofessional rounds can reinforce the importance of teamwork and provide ongoing support for collaborative practice. Ideally, interprofessional education initiatives should be introduced as early as medical school, laying the foundation for effective teamwork and communication strategies throughout a physician's career.

### 5.2 Professional hierarchy

The hierarchical nature of healthcare professions, where certain disciplines and specialties are viewed as more prestigious than others, hinders collaborative practices (78, 79). Surgeons are traditionally positioned at the top of this hierarchy, potentially fostering a culture where the knowledge and skills of other team members are undervalued (80, 81). In cardiothoracic surgery, this dynamic can be pronounced due to the complex procedures and critical nature of the diseases involved, which often necessitate rapid, decisive actions that may reinforce hierarchical decision-making. This environment can create considerable barriers to integrating other healthcare and administrative team members in planning and execution phases.

Addressing these hierarchical disparities requires cultural shifts within teams to value equally each member's contributions and promote shared responsibility in patient outcomes. Interprofessional communication frameworks aimed at reducing socio-hierarchical gaps should be introduced early in the education and training process. For example, the “TRI-O” guide of interprofessional communication skills, which emphasizes openness for collaboration, information, and discussion, has been shown to help undergraduate medical, nursing, and health nutrition students initiate partnership-based communication and mutual collaboration (82). Encouraging leadership roles among non-surgeon healthcare professionals and implementing mentorship programs can further balance power dynamics (83). Moreover, creating inclusive identities within cardiothoracic surgery teams can help all members view themselves as part of a larger, cohesive unit rather than separate, hierarchical entities. This can be achieved by emphasizing the value of each profession's contribution to patient care and integrating this perspective in the training and daily operations of the clinical team (84). Institutions should support these initiatives by embedding interprofessional collaboration in their culture and mission statements.

### 5.3 Professional stereotypes

Professional stereotypes further complicate interactions among diverse healthcare teams. For instance, surgeons and nurses often hold preconceived notions about each other's roles, competencies, and educational levels, which can lead to tensions and inefficient teamwork (85, 86). These stereotypes are typically formed during early educational experiences and are reinforced throughout professional practice (72). In cardiothoracic surgery, where stakes and stress levels are exceptionally high, professional stereotypes can severely disrupt the collaborative atmosphere needed to ensure patient safety and optimal outcomes. Surgeons might view nurses and allied health professionals merely as assistants rather than as integral contributors who can offer valuable insights into patient care.

Interprofessional education can dismantle these stereotypes by encouraging shared experiences and mutual learning. Educational programs should provide cardiothoracic surgery trainees with opportunities for contact with other healthcare professionals to overcome prejudice, invalidate negative attitudes, and foster favorable opinions toward other professions (87, 88). Collaborative problem-solving tasks can promote such intergroup contact, thereby reducing professional stereotypes (84). Leadership support is also important in this context; leaders should actively promote interprofessional education to help shift cultural norms and reduce resistance to change (86). Continuous professional development programs that provide practical training in interprofessional skills are valuable tools to sustain these efforts.

### 5.4 Resistance to role expansion

Interprofessional education often requires professionals to engage in roles that may traditionally fall outside their scope. This approach may involve surgeons participating in nursing tasks and vice versa, to foster a better understanding of each other's roles and develop mutual respect (89). However, such role expansion can be met with resistance due to fears of deskilling or overstepping professional boundaries (86). In cardiothoracic surgery, where tasks demand precision and specialization, this blending of roles can be challenging. Cardiothoracic surgeons may fear that engaging in traditionally nursing-centric tasks could dilute their specialized skills or undermine their authority in the multidisciplinary team. Conversely, nurses might be reluctant to perform surgical tasks or make decisions typically reserved for surgeons, fearing errors and legal repercussions (90).

Overcoming resistance to role expansion requires addressing concerns about deskilling and overstepping professional boundaries. One effective method is the use of role theory to help professionals understand and negotiate their roles within a team (91, 92). By clarifying role expectations and boundaries, healthcare professionals can better appreciate their colleagues’ contributions without feeling that their own skills are being undermined (93). Interprofessional education programs that include simulated scenarios can help cardiothoracic surgery trainees experience role expansion in a controlled and supportive environment (94, 95). These programs should be structured to demonstrate how role flexibility can improve patient outcomes (96). Furthermore, cardiothoracic surgeons should be encouraged to view role expansion as an opportunity for professional growth rather than a threat. Leadership support and institutional policies can play a significant role in promoting this perspective.

### 5.5 Logistical and administrative hurdles

Organizing interprofessional education initiatives in cardiothoracic surgery presents significant logistical and administrative challenges. Successful implementation of joint educational sessions requires coordination between multiple departments that operate on varying schedules and have different educational backgrounds and priorities. Moreover, cardiothoracic surgery involves lengthy procedures that demand precise timing and availability of various specialists, complicating the integration of interprofessional education into routine training timelines. Beyond scheduling, logistical hurdles may include allocating spaces that can accommodate multidisciplinary teams and providing resources, such as appropriately equipped simulation labs (97).

Overcoming these logistical and administrative challenges requires strong institutional leadership, organizational support, and strategic planning. Coordination across departments can be facilitated by the establishment of a dedicated, adequately resourced, centralized faculty workgroup (98). This workgroup should include representatives from cardiothoracic surgery, anesthesia, nursing, and other relevant disciplines to ensure integration of all perspectives into the planning process. Furthermore, aligning academic calendars and creating uniform scheduling blocks specifically for interprofessional education activities can mitigate the difficulties posed by different schedules among disciplines (99). Investment in shared facilities, such as simulation labs and collaborative learning spaces, can address spatial challenges by providing dedicated venues for interprofessional education (98). To sustain these efforts, institutions should provide ongoing logistical, administrative, and financial support.

## 6 Discussion

Deep integration of interprofessional education in cardiothoracic surgery presents significant challenges but holds considerable potential to improve patient care, reshape professional relationships, and transform the overall culture of this specialty. Successful implementation, however, requires careful consideration of individual, organizational, and systemic factors. By examining the current landscape and emerging trends, we can better understand how the future of cardiothoracic surgery may be influenced by our ability to navigate this educational evolution.

Redesigning cardiothoracic surgery curricula with a greater emphasis on interprofessional education necessitates significant revisions to current training programs. These changes must extend beyond simply adding new content; they require a paradigm shift in how we conceptualize surgical education. Future programs must seamlessly integrate interprofessional competencies throughout, recognizing them as core components of professional certification rather than optional add-ons. This goal can be achieved through structured programs that combine various training and educational approaches, while offering diverse professional perspectives on patient care. It is paramount that these new pedagogical elements preserve the depth of specialized knowledge that cardiothoracic surgeons must possess.

This path to integration may require approval from multiple accreditation bodies and licensure organizations, especially in countries where a central regulatory authority is lacking or has diminished power (100, 101). The unique requirements and expectations of each profession's governing body can make alignment a delicate and time-consuming process (102). Success depends on close collaboration among these agencies to develop a harmonized framework that supports interprofessional education while maintaining the rigorous standards of each specialty.

Concrete and measurable interprofessional education outcomes will provide accreditation bodies with a strong mandate to endorse and sustain these programs. However, current metrics for interprofessional education often fall short, failing to capture subtle improvements in team dynamics and patient outcomes (103). In cardiothoracic surgery, where outcomes are closely linked to patient survival and quality of life, developing specific indicators that accurately reflect the effectiveness of interprofessional collaboration is crucial. These indicators should incorporate reliable and valid tools for measuring team functionality and performance. Furthermore, they must assess tangible impacts on patient care, such as recovery times, postoperative morbidity rates, and patient satisfaction. When selecting, developing, and validating evaluation tools for interprofessional education programs in cardiothoracic surgery, it is crucial to consider the specific learning objectives, the stage of learners’ professional development, and the contextual factors of the surgical environment.

Building on the need for robust evaluation of interprofessional collaboration initiatives, several validated tools have been developed and adapted for use in healthcare education settings. Among these, the Attitudes Toward Health Care Teams Scale (ATHCTS) has shown promise for evaluating interprofessional teamwork attitudes. Depending on the specific model, the ATHCTS may consist of various subscales, including quality of care (delivery), time constraints, physician centrality, team efficiency, and patient-centered care (104–106). It has shown acceptable to good internal consistency for its subscales and has been validated with both undergraduate and graduate healthcare students (107). Another widely used measure for assessing interprofessional education is the Readiness for Interprofessional Learning Scale (RIPLS) (108–111). However, recent critiques have highlighted psychometric concerns with the RIPLS, including issues with its factor structure and internal consistency, suggesting careful consideration of its use (112). The Interdisciplinary Education Perception Scale (IEPS) was identified as the most frequently used outcome measure in a systematic review assessing the efficacy of interprofessional education activities during student clinical placements (113). The IEPS assesses perceptions of interprofessional collaboration through four subscales: competency and autonomy, perceived need for cooperation, perception of actual cooperation, and understanding of others’ roles (114). This scale has demonstrated good psychometric properties, with Cronbach's alpha values generally exceeding 0.80 and test-retest weighted kappa values ranging from fair to moderate across its various subscales (115–119). This instrument could potentially be adapted to address the specific needs of cardiothoracic surgery. A validated, specialty-specific tool could enable precise and meaningful assessment of interprofessional education initiatives, thereby contributing to the development of evidence-based practices.

The success of interprofessional education in cardiothoracic surgery also hinges on the ability of clinical and educational supervisors to facilitate this new approach. Faculty development should aim to equip instructors with the skills necessary for interprofessional education, including conflict resolution and collaborative leadership (97, 120, 121). Additionally, educators must learn to create learning environments that authentically reflect the high-pressure and time-sensitive scenarios that are common in the field. By leveraging experiential learning and interactive teaching methods, interprofessional competencies can be readily integrated into the intensive technical training that characterizes cardiothoracic surgery (122, 123).

As faculty members advance to leadership roles, it is important that they actively promote interprofessional education practices as *a priori*ty within their institutions (89). This requires a proactive approach to overcome resistance stemming from traditional practices that favor professional boundaries in cardiothoracic surgery. Leaders should not only provide opportunities for interprofessional learning but also drive cultural shifts toward more collaborative practices. They must also engage influential stakeholders to secure the necessary resources for these initiatives (81). For example, administrators and managers who determine educational policies and control resources can implement changes in course structures, provide faculty support through academic incentives, and allocate funding to operate interprofessional education budgets (124–126).

Political and financial support at the government level can also create essential incentives for organizations to prioritize interprofessional education activities (127). Policies should encourage the adoption of interprofessional education by embedding it within accreditation standards and professional development requirements for cardiothoracic surgeons. Additionally, the procurement and maintenance of financial support for interprofessional education initiatives should be based on realistic budgets and detailed business plans (126, 128). For instance, funding could be allocated for developing interprofessional simulation centers that provide practical experience in a multiprofessional setting. Sharing these simulation centers and other resources, such as administrative support, can help reduce costs and ensure the long-term sustainability of interprofessional education initiatives.

Simulation centers undoubtedly offer valuable training opportunities; however, their effectiveness in interprofessional education depends on careful scenario design and facilitation. Scenarios that deliberately incorporate role ambiguity or conflicting priorities among team members can stimulate rich discussions about professional boundaries and collaborative decision-making. Additionally, involving patients or patient advocates in the design and execution of simulations can provide unique insights into team dynamics from the patient's perspective (129). This approach not only improves the realism of scenarios but also reinforces patient-centered care principles (130).

Complementing simulation-based approaches, artificial intelligence (AI) and machine learning are emerging as valuable tools in improving nontechnical skills and interprofessional education (131). AI-powered platforms can analyze team interactions during simulated scenarios, providing objective feedback on communication patterns, leadership dynamics, and decision-making processes (132). Machine learning algorithms can process complex team performance data to identify effective collaboration strategies and areas for improvement in interprofessional settings. These technologies may also facilitate adaptive learning experiences, automatically adjusting scenario difficulty based on team performance to optimize learning outcomes (133). However, integrating AI and machine learning into interprofessional education faces several challenges. These include ensuring that AI-based assessments capture the nuances of human interactions, avoiding the reinforcement of biases in team dynamics and maintaining the authenticity of interpersonal skill development. Additionally, the high cost and technical expertise required for implementation may limit widespread adoption, particularly in resource-constrained settings.

While the role of interprofessional education is usually discussed within the context of training and accreditation, it is worth considering its influence on the hiring process for cardiothoracic surgeons. Traditional job interviews often fall short in evaluating a candidate's ability to work effectively in interprofessional teams (134). In response, some institutions have implemented simulated interprofessional scenarios or team-based interviews into their recruitment process. These methods aim to gauge a candidate's collaborative competencies and their potential to contribute to a culture of interprofessional practice. However, it is important to recognize that these initial assessments are just a starting point. The complex and evolving nature of cardiothoracic surgery demands continuous interprofessional education throughout a surgeon's career to refine and adapt these essential collaborative skills.

**Figure 1:**
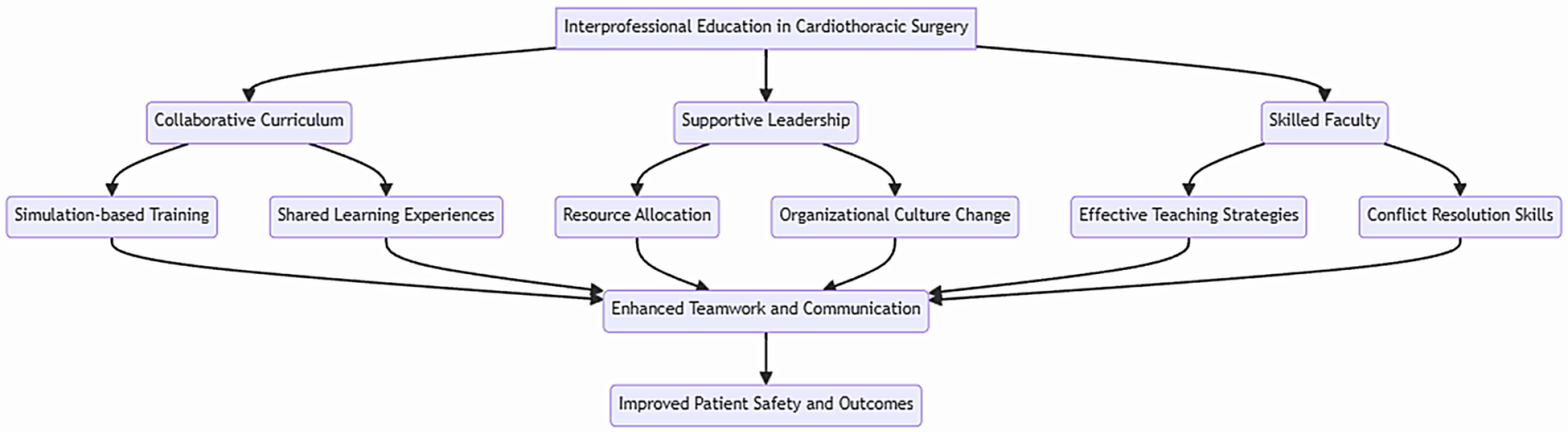
Interprofessional education framework in cardiothoracic surgery.

This narrative review possesses certain strengths. First, it is one of the first comprehensive examinations on interprofessional education in cardiothoracic surgery, providing an overview of existing practices, outcomes, and future directions. By synthesizing evidence from a wide range of sources, this review offers a broad perspective and a conceptual framework for interprofessional education in cardiothoracic surgery (Figure 1). Second, it provides practical insights into strategies for successful implementation, such as the use of high-fidelity simulation and the development of interprofessional outcome measures. Third, the review identifies key gaps in the literature and suggests directions for future research. This can guide the development of more rigorous and targeted studies to evaluate the effectiveness of interprofessional education interventions in cardiothoracic surgery. Finally, the timeliness and relevance of this review are significant. With an increasing emphasis on collaborative practice and patient-centered care, interprofessional education has become a critical component of modern surgical training. As such, this review is likely to be of interest to a wide range of stakeholders, including educators, clinicians, researchers, and policymakers.

Despite its strengths, this literature review has several limitations that should be acknowledged. As a narrative review, it lacks the methodological rigor of a systematic review. The literature search was not exhaustive, and the selection of studies was not based on strict criteria. Consequently, relevant studies may have been overlooked, and the review may be subject to selection bias. Furthermore, the quality of the included studies was not formally assessed, limiting the ability to draw firm conclusions based on their findings. Another limitation is the paucity of high-quality studies investigating interprofessional education in cardiothoracic surgery. Most of the included studies were descriptive and had small sample sizes, reflecting the relative infancy of research in this area. Moreover, the included studies exhibited substantial heterogeneity in terms of intervention types, study populations, and outcome measures. This heterogeneity makes it challenging to compare results across studies and draw generalizable conclusions. Additionally, the included studies were conducted in high-income countries, particularly in North America and Europe. This geographical bias limits the applicability of the findings to other healthcare settings, especially in low- and middle-income countries, where the challenges to implementing interprofessional education may differ significantly. Finally, this review did not explore the cost-effectiveness of interprofessional education interventions. Given the resource-intensive nature of some of these interventions, such as high-fidelity simulation training, it is important to consider their financial implications and whether the benefits justify the costs. Future studies should include economic evaluations to guide decision-making about the allocation of resources for interprofessional education in cardiothoracic surgery.

## 7 Conclusions

Interprofessional education holds great promise for improving patient outcomes and professional satisfaction in cardiothoracic surgery. Realizing this potential necessitates extensive curricular reforms, faculty development initiatives, strong leadership support, and sustained political and financial commitment. Furthermore, ongoing research is warranted to develop validated assessment tools and standardized outcome measures that will enable the evaluation and continuous improvement of interprofessional education programs across different healthcare contexts. By addressing these key areas, the field of cardiothoracic surgery can make significant progress toward harnessing the full benefits of interprofessional education, ultimately improving patient care quality and outcomes.
